# Biosensors and Smart Analytical Systems in Food Quality and Safety: Status and Perspectives

**DOI:** 10.3390/foods12122292

**Published:** 2023-06-07

**Authors:** Barbara Giussani, Jordi Riu

**Affiliations:** 1 Dipartimento di Scienza e Alta Tecnologia, Università degli Studi dell’Insubria, Via Valleggio 9, 22100 Como, Italy; 2 Department of Analytical Chemistry and Organic Chemistry, Universitat Rovira i Virgili, 43007 Tarragona, Spain; jordi.riu@urv.cat

The primary focus of research in food production revolves around ensuring food quality and safety. Consequently, there is a significant interest in establishing dependable approaches to detect, identify, measure, describe, and monitor any quality and safety concerns that may arise in food production. To enhance the efficiency of production processes and maintain high-quality standards, the food industry also requires tools that can minimize waste, promptly identify potential process issues, and reduce the need for extensive laboratory analysis. These tools aim to streamline operations and reduce the workforce dedicated to such tasks.

This Special Issue brings together new strategies and applications that address these needs in the food industry. The development of new analytical sensors or new analytical methods that can be used in the field, if possible, using as few solvents as possible (ideally, no solvents at all) and analysing the sample as it is, without or with minimal pre-treatment, was the primary focus of this Special Issue. The optimisation of reagentless methods with no or minimal pre-treatment, also leads to the advantage that that method, suitably adapted, may also be successfully used for the direct control in the production process itself.

The abundance of literature on the subject reinforces the fact that spectroscopic techniques are highly suitable for such applications. The availability of affordable and portable spectroscopic instruments has the potential to bring significant advantages in food quality control. NIR (near infrared) and Raman techniques are the most widely used, with a large prevalence of applications in the NIR range. Virtually all the applications involving spectroscopic instrumentation require multivariate data processing to extract the relevant information from the spectra, very often starting from principal component analysis (PCA) as a method of data visualisation to multivariate regression techniques (almost always PLSR—partial least square regression) or classification techniques, depending on the problem to be solved. The methods used are mainly linear, except in some specific cases where non-linear methods perform better, as is evident from the articles in this collection.

Jean Frederic Isingizwe Nturambirwe and co-authors [1] conducted a feasibility study to classify bruise damage in three apple cultivars using a non-contact spectrometer working in the range of 800–2500 nm. Six machine learning classification algorithms were tested together with two feature selection methods to determine the most informative wavelengths for classification. The results were very encouraging, with precision values ranging from 0.7 to 0.9 depending on the cultivar. Elena Cazzaniga and co-authors [2] determined the lipid content of hazelnuts from various regions of origin, including Italy, Chile, Turkey, Georgia, and Azerbaijan, using two NIR instruments: a benchtop FT-NIR spectrometer equipped with an integrating sphere and an optic fibre probe, and a pocket-sized and battery-powered miniaturized device. PLSR was used as the regression method. In this case, the portable sensor gave lower performances if compared to the benchtop one: the probe yielded the most favourable outcomes. The use of spectroscopic methods in combination with chemometrics also proved successful for Putthiporn Khongkaew and colleagues [3], who determined the curcuminoids in turmeric with both benchtop and portable NIR and Raman spectrometers. The five proposed methods using different instrumentation (FT-IR, Raman, and NIR) were compared in terms of precision and accuracy: the results indicated a strong agreement between the benchtop and hand-held methods, demonstrating no noteworthy differences between them. Portable Raman spectroscopy was also used by Elisa Robotti and colleagues [4] to study illicit conservation treatments to extend the shelf life of fresh fish. Partial least square discriminant analysis (PLS-DA) was able to correctly classify samples treated with “Cafodos” adulterant (a blend of citric acid, sodium citrate, and hydrogen peroxide—which is prohibited in fish products—that enhance the lustre of fish skin and extend its shelf life by means of a bactericidal effect) from untreated samples both in fish muscle and skin.

Compact solids are often analysed with miniaturized instruments. However, when it comes to analysing liquids, researchers often need to develop strategies specifically tailored to this objective. As an example, Giussani and co-workers [5] demonstrated resourcefulness by employing low-cost portable NIR instrumentation to analyse edible oil. In their research paper, they developed a home-made cell that operates through transflectance measurements. This innovative approach enabled them to successfully analyse and classify oil samples of various types.

A very interesting application was presented also by Zhiyang (Stan) Tu [6] and co-workers. Their study aimed to enhance the understanding of using NIR spectroscopy in the analysis of spray-dried samples and to show potential for their real-time characterization, since process analysis of food products represents another major challenge for modern analytical chemistry. The article involved a comparison of two different sample preparation methods and two spectrometers. The study also investigated the influence of sample preparation strategies on the development of NIR calibration models. The validation of the usability of a portable NIR spectrometer was conducted by predicting the moisture content of spray-dried powders measured through both a glass container and a fibre optic cable, with good results. The study proposed by Daniele Tanzilli and co-authors [7] aimed at investigating the feasibility of implementing online monitoring for a pesto sauce production process using NIR spectroscopy and chemometric tools. The spectra of an intermediate product were acquired in real-time using a NIR probe installed directly on the process line. PCA was successfully employed for exploratory data analysis and to construct multivariate statistical process control (MSPC) charts. Additionally, PLSR was utilized to develop real-time prediction models for two distinct quality parameters of pesto sauce.

As the evidence supporting the effectiveness of miniaturized NIR instruments in food-related applications continues to accumulate, there is a growing number of end-users adopting these instruments, partly due to their affordability. While considerable attention is given to the analytical protocol, including sampling, data collection, and data processing, the significance of investigating errors in raw data is often overlooked. Giulia Gorla and co-authors [8] proposed a chemometric method based on the study of error covariance matrices (ECMs), useful for studying uncertainty not only in data produced by miniaturised instruments but in spectroscopic instruments in general, using sugar in different packaging forms as the case study. The results showed that the impact of different sources of variability on measurement errors was contingent upon the characteristics of the sample and sensor. Therefore, it is essential to assess measurement errors prior to optimizing any analytical method.

Undoubtedly, one of the most extensively studied samples globally is milk, encompassing both commercial food products and breast milk. In this collection of articles, as many as three out of fourteen (more than 20%) are dedicated to milk analysis. Christopher Karim Akhgar and colleagues [9] proposed a novel method based on mid-infrared spectroscopy to predict concentrations of individual fatty acids as well as key sum parameters in human milk. The acquired spectra were used to build a PLSR model, using gas chromatography-mass spectrometry (GC-MS) as reference data. An evaluation of the environmental impact of the proposed method demonstrated its clear superiority over GC-MS methods. Consequently, the proposed method offered a high-throughput, environmentally friendly alternative to conventional resource-intensive techniques. Breast milk was also analysed by Ziqian Ye and co-authors [10], in their case for the detection of traces of amoxicillin: antibiotic residues can have an impact on the intestinal flora and health of babies. In this case, however, spectroscopy was not employed: a system based on a combination of colorimetric methods and image pre-processing with artificial intelligence and back-propagation artificial neural networks (BP-ANN) was proposed. The colorimetric method employed in this study involved the utilization of gold nanoparticles in combination with single-strand DNA aptamers. By introducing various concentrations of amoxicillin, distinct colour outcomes were produced. The resulting colour image was then captured using a portable image acquisition device. BP-ANN data elaboration allowed to obtain good linear correlations between the colorimetric image value and concentration of target amoxicillin. To monitor commercial milk freshness, an intelligent label was developed by Ruoting Liu and colleagues [11]. The change in freshness was, therefore, correlated with a change in the sensor colour. A cellulose-based matrix was developed using rice straw particles ranging in size from 0.125 to 0.150 mm. The matrix was bis-quaternised to attach bromocresol purple to detect changes in pH values of 5.2–6.8, as pH is often used to assess milk freshness. The intelligent label exhibited an excellent response. Colour change was also used as an analytical strategy using aptamer-functionalised nanobeads by Yu Chen and co-workers [12], who developed an aptasensor for the determination of aflatoxin B1 in cereal samples, an analyte of great concern due to its toxicity. Two other very toxic molecules present in fish were the subject of the review proposed by Jaume Reverté and co-authors [13], while zearalenone was analysed in edible and medicinal coix seeds by Kaiyi Guan and co-workers, using a strategy based on an indirect competitive enzyme-linked immunosorbent assay (ic-ELISA) [14]. This method was applied to the determination of zearalenone in 122 batch coix seeds from different parts of China, showing that the occurrence of zearalenone is a universal problem in various districts in China.

Finally, Yanan Xia and co-workers studied the variation and correlation of different flavour parameters and bacterial diversity of low-salt hotpot sauce during storage [15]. Multivariate statistical methods such as cluster analysis (CA), principal component analysis (PCA), or multivariate analysis of variance (MANOVA) were applied to the investigation of changes in the physicochemical quality, flavour, and bacterial diversity of hotpot sauces with different salt concentrations. The results showed that the quality of hotpot sauce deteriorated gradually with storage, finding the right storage conditions to reduce spoilage.
